# Artificial Intelligence for Personalized Prediction of Post-TIPS Outcomes: Integrating Clinical, Biochemical, and Radiomics Data—A Narrative Review

**DOI:** 10.3390/jcm15166211

**Published:** 2026-08-11

**Authors:** Alessio Barrancotto, Simone Di Cola, Fabio Melandro, Lucia Lapenna, Anthony Vignone, Antonio Bencivenga, Arianna Brancati, Pierleone Lucatelli, Mario Corona, Stefania Gioia, Silvia Nardelli

**Affiliations:** 1 Department of Translational and Precision Medicine, Sapienza University of Rome, 00185 Rome, Italy; alessio.barrancotto@uniroma1.it (A.B.); simone.dicola@uniroma1.it (S.D.C.); lucia.lapenna@uniroma1.it (L.L.); anthony.vignone@uniroma1.it (A.V.); antonio.bencivenga@uniroma1.it (A.B.); arianna.brancati@uniroma1.it (A.B.); stefania.gioia@uniroma1.it (S.G.); 2 Department of General Surgery and Surgical Specialties Paride Stefanini, Sapienza University of Rome, 00185 Rome, Italy; fabio.melandro@uniroma1.it; 3 Department of Radiological Sciences, Oncology and Pathology, Sapienza University of Rome, 00185 Rome, Italy; pierleone.lucatelli@gmail.com (P.L.); m.corona@policlinicoumberto1.it (M.C.)

**Keywords:** TIPS, hepatic encephalopathy, artificial intelligence, machine learning, radiomics, risk prediction, body composition, multimodal prediction, explainable AI

## Abstract

Transjugular intrahepatic portosystemic shunt (TIPS) is an established treatment for complications of portal hypertension, but hepatic encephalopathy (HE), liver dysfunction, rebleeding, and mortality remain difficult to predict in otherwise eligible candidates. However, predicting post-TIPS outcomes remains challenging using conventional risk scores such as MELD 3.0 and Child–Turcotte–Pugh. This narrative review critically evaluates how clinical, biochemical, procedural, conventional imaging, handcrafted radiomics, and deep-learning features can be integrated for personalized post-TIPS risk prediction, supplemented by backward and forward reference checking. Thirty-two original post-TIPS studies met the core inclusion criteria: 18 focused primarily on clinical, biochemical, hemodynamic, microbiome, or procedural predictors and 14 on imaging, body composition, radiomics, or multimodal models. AI, ML, and radiomics models, by the aim of logistic regression, tree-based ensembles, support vector machines, artificial neural networks, and hybrid deep-learning models, able to capture non-linear interactions, have consistently demonstrated improved predictive performance compared with conventional scores, particularly for HE, with reported incidences of approximately 20–47%, mortality, and liver dysfunction. Their advantage lies in the ability to model complex, non-linear relationships and integrate heterogeneous data sources, including laboratory parameters, ammonia levels, hemodynamic variables, and imaging-derived features. Radiomics and deep learning approaches further enhance predictive accuracy. CT is currently the principal imaging substrate, while direct post-TIPS radiomics evidence for MRI and ultrasound remains sparse. Studies of liver and spleen morphology, portal-vein geometry, muscle and adipose tissue, and radiomic texture suggest incremental information beyond conventional scores, particularly when clinical and imaging features are combined; however, negative volumetric findings show that additional image features do not automatically improve prediction. However, most studies are retrospective, single-center, and lack external validation and no validated transformer-based or other sequence model has yet been established for post-TIPS outcomes. Standardization issues in radiomics and limited model interpretability remain significant barriers. Future directions should lead to prospective multicenter cohort validation, increasing sample sizes, harmonized imaging and endpoint definitions, locked external validation with recalibration, and the development of clinically interpretable tools to make it easier to identify those patients suitable for TIPS and their post-procedural management.

## 1. Introduction

Transjugular intrahepatic portosystemic shunt (TIPS) is a well-established therapeutic option for managing complications of portal hypertension, particularly refractory ascites and variceal bleeding [1,2], by creating a low-resistance conduit between the portal and systemic venous systems through the placement of a covered stent, effectively reducing portal pressure [2]. Due to the significant haemodynamic and metabolic changes that TIPS provides, not all patients can be considered suitable candidates for its placement [3]. Therefore, careful patient selection is necessary, and it should be standard clinical practice to screen all patients prior to TIPS placement using specific physical, biochemical, and radiological examinations [4]. Contemporary guidance emphasizes multidisciplinary assessment of liver reserve, cardiac and pulmonary function, infection, neurological vulnerability, vascular anatomy, indication, and procedural strategy before TIPS placement. Even after recognized contraindications have been excluded, substantial residual heterogeneity remains in the probability of post-TIPS HE, liver dysfunction, further decompensation, rebleeding, and death [5,6,7,8].

Several prognostic scores have been developed to estimate post-TIPS outcomes, including the Child–Turcotte–Pugh (CTP), Freiburg Index of Post-TIPS Survival (FIPS), and Model for End-Stage Liver Disease (MELD 3.0) [9,10]. While these tools are widely used, they do not fully represent cognition, frailty, portal-flow architecture, body composition, inflammatory or metabolic phenotypes, or high-dimensional imaging, and are limited by their reliance on static, linear relationships and their inability to incorporate complex patient-specific variables or advanced imaging data.

In this context, artificial intelligence (AI) and machine learning (ML) have emerged as promising tools for improving risk stratification by enabling multidimensional and personalized prediction models [11].

The present review is designed to provide a balanced and clinically oriented synthesis of clinical, biochemical, procedural, conventional imaging, handcrafted radiomics, deep radiomics, and explainability across multiple post-TIPS outcomes. Its novel contribution is the explicit comparison of these data domains, a complete radiomics workflow, a modality-specific appraisal of CT, MRI, and ultrasound, and a translation-readiness assessment that distinguishes discrimination from external validity and actionable clinical benefit [12,13].

The objectives are to: (i) define the clinical prediction tasks that follow TIPS; (ii) distinguish AI, machine learning (ML), deep learning (DL), handcrafted radiomics, and deep radiomics; (iii) critically compare model families and data domains; (iv) evaluate reproducibility, interpretability, and validation; and (v) propose a research agenda for clinically deployable multimodal prediction.

### Narrative Search Strategy and Study Selection

A systematic search of PubMed/MEDLINE was performed through 20 June 2026. The search combined terms for the procedure (“transjugular intrahepatic portosystemic shunt”, “TIPS”, and “TIPSS”) with terms for outcomes (“hepatic encephalopathy”, “mor-tality”, “survival”, “liver failure”, “liver dysfunction”, “rebleeding”, “ascites”, “decompensation”, and “hemodynamic”) and prediction methods or data types (“prediction model”, “nomogram”, “machine learning”, “artificial intelligence”, “deep learning”, “radiomics”, “texture”, “body composition”, “computed tomography”, “magnetic resonance”, “ultrasound”, “microbiome”, and “metabolomics”). Reference lists of included studies, relevant guidelines, and previous reviews were also examined to identify additional original investigations. Thus, eligible studies included adult patients after TIPS and investigated predictors or predictive models designed to estimate post-TIPS outcomes at the individual patient level.

Reviews, editorials, conference abstracts without sufficient data, case reports, non-TIPS cohorts, purely technical segmentation studies, and studies without a post-TIPS outcome were excluded from the core evidence set. Studies of MRI or ultrasound in cirrhosis without a direct post-TIPS prediction endpoint were used only to clarify modality opportunities and were explicitly labeled as contextual evidence.

Thirty-two original post-TIPS studies met the core inclusion criteria and form the structured evidence synthesis below: 18 clinical, biochemical, procedural, hemodynamic, microbiome, or metabolomic studies (Table 1) and 14 imaging, body-composition, radiomics, or multimodal studies (Table 2). Guidelines, reporting standards, methodological papers, and prior reviews were used for context but were not counted among the 32 original studies. Because this was a narrative rather than systematic review, no meta-analysis was undertaken; methodological quality was appraised qualitatively using principles from PROBAST+AI, TRIPOD+AI, CLAIM, CLEAR, RQS, and IBSI [14,15,16,17,18,19].

**Figure 1:**
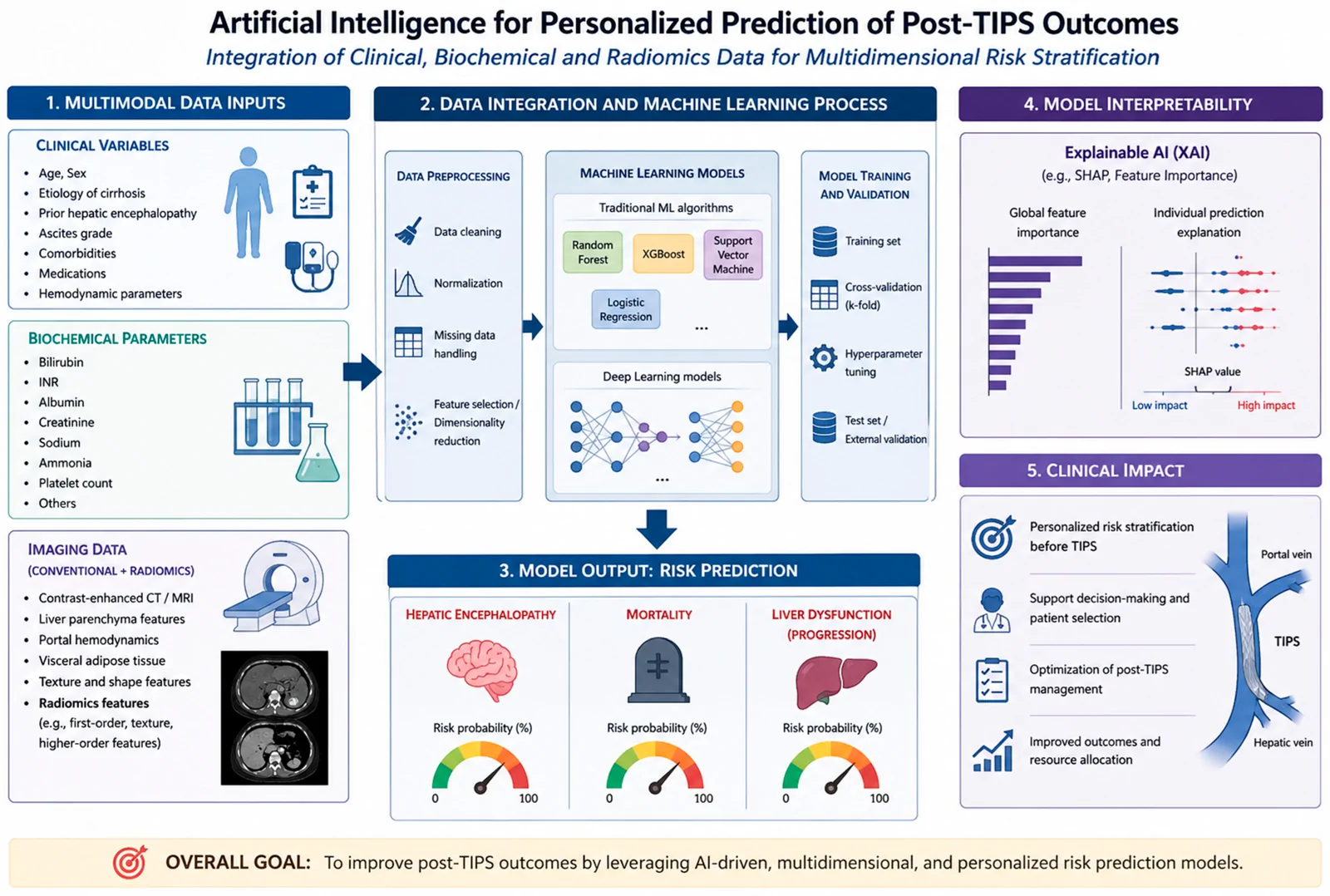
Conceptual framework for multimodal, explainable prediction of post-TIPS outcomes. Clinical, biochemical, procedural, and imaging/radiomics data are preprocessed and integrated in statistical-learning, machine-learning, or deep-learning models. Outputs should be calibrated to specific outcomes and time horizons, interpreted with transparent methods, and linked to actionable clinical pathways. AI tools were used for image generation.

A conceptual framework is schematically illustrated in Figure 1.

## 2. Transjugular Intrahepatic Portosystemic Shunt (TIPS): Indications, Contraindications, and Prediction of Post-TIPS Outcomes Targets

### 2.1. Indication and Contraindication Screening Versus Residual-Risk Prediction

TIPS is one of the main therapies for complications of portal hypertension, such as refractory ascites, gastro-oesophageal varices, and variceal bleeding, in both cirrhotic and non-cirrhotic patients [2,52].

The main goal of TIPS is to create a low-resistance channel by placing a covered metallic stent between the portal venous system and the systemic venous system, bypassing the liver, in order to reduce portal hypertension and its complications and control portal hypertension-related bleeding and ascites in appropriately selected patients [6,53]. Due to these significant haemodynamic and metabolic changes that TIPS provides, careful patient selection is necessary, and it should be standard clinical practice to screen all patients with specific physical, biochemical, and radiological examinations before undergoing TIPS placement [54].

According to recent guidelines [1,52,55], the main contraindications are acute or chronic heart failure, severe pulmonary hypertension (PAPs > 45–50 mmHg), refractory or previous severe episodes of hepatic encephalopathy, and advanced end-stage liver disease (Child-Pugh class C and/or MELD-Na > 15 according to traditional scores). Therefore, accurate patient selection is mandatory to reduce post-TIPS complications, the most frequent of which are hepatic encephalopathy, liver failure, and death.

Current guidance does not support a single laboratory cut-off as an absolute decision rule for all TIPS indications. Instead, clinicians integrate the urgency and expected benefit of TIPS with severe uncontrolled HE, advanced hepatic dysfunction, active infection, major cardiac or pulmonary disease, anatomical feasibility, and transplant candidacy. These assessments are primarily diagnostic and eligibility decisions. A risk model becomes relevant only after this first gate, when uncertainty remains among patients for whom TIPS is still being considered [5,6,7].

Although TIPS can be considered a therapy to improve the survival rate and quality of life of selected patients with clinically relevant portal hypertension, TIPS placement can lead to complications that significantly reduce survival rates, particularly within the first three to six months after placement [20,56].

The clinically relevant objective is therefore not limited to identifying whether a patient meets predefined contraindication criteria, but rather to estimating, among eligible or borderline candidates, the probability of specific adverse outcomes over a defined time horizon, the uncertainty associated with these estimates, and the potential impact on clinical decision-making. This framework avoids the development of circular models that merely replicate existing exclusion criteria and promotes the assessment of incremental predictive value beyond routine clinical judgment.

### 2.2. Incidence and Clinical Impact of Post-TIPS Outcomes

HE is the most frequently modeled post-TIPS complication. Across a recent systematic review of 24 studies and 5197 patients [12], the reported incidence ranged from 19.9% to 46.6%, reflecting differences in case mix, prophylaxis, stent strategy, outcome definition, and follow-up. OHE can lead to hospital admission, loss of independence, impaired quality of life, shunt reduction or occlusion, and delayed transplant assessment. It should therefore be modelled as a time-to-first event, recurrent event, severity-specific endpoint, or patient-centered burden rather than as an undifferentiated binary label whenever data permit [12].

Additional relevant post-TIPS outcomes include mortality and liver dysfunction. Early mortality may reflect loss of hepatic reserve, renal dysfunction, infection, cardiac decompensation, or excessive portal decompression, whereas later mortality is influenced by transplantation, recurrent decompensation, malignancy, and the underlying trajectory of cirrhosis. A large multicenter cohort found that post-TIPS OHE was associated with long-term rather than short-term mortality, illustrating why outcome horizon and competing risks must be specified [37,57].

Other relevant targets include control of ascites or bleeding, variceal rebleeding, further decompensation, shunt dysfunction, and hemodynamic response, even if these outcomes can compete: aggressive decompression may improve bleeding control while increasing HE risk. In a multicenter study of pre-emptive TIPS, 8 mm stents reduced one-year OHE without compromising rebleeding control, and a post-TIPS pressure-gradient range of approximately 7–13 mmHg was associated with a more favorable balance. Prediction should therefore support multi-outcome benefit-risk decisions rather than optimize one endpoint in isolation [58,59].

### 2.3. Traditional Scores and Eligible Candidate Predictors

To identify patients at higher risk of mortality after TIPS placement, validated scores were developed based on clinical and biochemical factors.

The Child–Turcotte–Pugh (CTP) score [60,61], which is based on serum albumin, total serum bilirubin, international normalized ratio (INR), ascites grade, and encephalopathy grade, classifies patients into three classes: A (score 5–6), B (score 7–9), and C (score 10–15). Patients with a CP score of C are considered at high risk of TIPS placement.

The Freiburg Index of Post-TIPS Survival (FIPS) [56,62] score takes into account age, serum creatinine, albumin, and bilirubin. Patients with a FIPS score greater than 0.92 are considered to be at high risk of complications and mortality following TIPS [10,57,63,64].

Currently, the most accurate traditional score for predicting outcomes following TIPS is the Model for End-Stage Liver Disease (MELD) score, which was recently refined to MELD 3.0 [10,65]. This score is based on an evaluation of patient gender, international normalized ratio (INR), serum sodium, creatinine, albumin, and total bilirubin. A recent meta-analysis involving 855 patients demonstrated MELD 3.0 concordance indices (C-indices) of 0.727 and 0.713, respectively, at three- and six-months post-TIPS. MELD 3.0 was shown to be superior to the Child-Pugh (CP) score and the Freiburg index of post-TIPS survival (FIPS) score in predicting mortality up to two years post-TIPS [10,65].

Nevertheless, all the above-mentioned scores have some limitations [66]. Firstly, these models are based on algorithms that define a static, linear mortality and complication rate. They do not involve any other patient-specific factors and do not allow for a multidimensional evaluation of risk. This is particularly important in the era of precision medicine, when therapeutic decisions are tailored to the individual patient. Technological progress and the implementation of innovations in first- and second-level instrumental diagnostics (e.g., radiomics) have provided an increasingly detailed knowledge of pathological features, which represent a significant part of comprehensive patient assessment in multidimensional evaluation.

## 3. Role of Artificial Intelligence (AI) and Machine Learning (ML) in the Creation of Integrated, Multidimensional, and Patient-Tailored Outcome Models

### 3.1. Distinguishing AI, ML, DL, Radiomics, and Model Families

Artificial intelligence (AI) and machine learning (ML) have emerged as promising tools for improving risk stratification by enabling multidimensional and personalized prediction models [67,68].

Artificial intelligence (AI) is an informatics discipline whose main goal is to create software with the ability to perform actions through a complex system of algorithms that require reasoning and data analysis. Its role is to classify results and make decisions that are useful in clinical practice and evidence-based medicine [69].

Machine learning is a subcategory of AI based on techniques that enable computers to learn from data using algorithms that can build statistical models to predict outcomes [70,71]. Logistic regression, penalized regression, random forests, gradient boosting, support vector machines, and neural networks can all be used as supervised ML methods. Logistic and Cox regression provide coefficients, familiar effect measures, and relatively stable performance in modest samples when assumptions and shrinkage are appropriate, reducing the possibility of overfitting in correlated feature sets. Tree ensembles capture interactions and non-linear thresholds but can be unstable in small datasets and require careful tuning and calibration. Support vector machines can perform well in high-dimensional spaces but do not natively produce calibrated probabilities. Neural networks are flexible but data-hungry [16,17,72].

Deep learning plays a central role in this field: using artificial neural links to create predictive tools, it can learn hierarchical representations directly from images, sequences, or other high-dimensional inputs [24,72,73,74].

Radiomics is a field of medical imaging that involves extracting and analyzing quantitative features of a specific segmented region of interest from diagnostic imaging scans [75,76], then converted into data that can be correlated with diagnosis, prognosis, and treatment response [42,75,77]. Handcrafted radiomics computes predefined first-order, shape, and texture features, such as voxel intensity, shape, internal heterogeneity, and complex, multi-scale patterns, from a segmented region of interest; deep radiomics or deep features are learned automatically by a neural network. Either representation can subsequently be combined with clinical variables in logistic regression, support vector machines, tree ensembles, neural networks, or other models [15,76,78].

### 3.2. Evidence from Clinical, Biochemical, Procedural Models, and Explainable AI

Early post-TIPS studies developed interpretable nomograms or risk scores from age, liver and renal function, sodium, ammonia, diabetes, stent diameter, and pressure-gradient reduction. Their value lies in accessible variables and transparent clinical logic, but apparent performance can be inflated by random split-sample validation, predictor selection before resampling, dichotomization, and low event-per-parameter ratios [21,22,23,28].

In recent years, ML has played an increasingly important role in predicting outcomes after TIPS, surpassing traditional prognostic scores based on linear, static relationships. In particular, ML’s ability to integrate clinical and biochemical variables allows for an accurate and personalized risk assessment. Zhong et al. [24] developed algorithms to predict post-TIPS complications (OHE at 3 months, liver dysfunction at 3 months, and 1-year mortality following early TIPS placement) in a sample of 207 patients with the aid of three different machine learning models based on Artificial Neural Networks (ANNs), whose accuracy was estimated by the analysis of the area under the curve (AUC). OHE occurred in 33 patients (15.9%) and the independent predictors were Albumin-Bilirubin Index (ALBI) grade (score 1, 2 or 3), age > 65 years, gender, and alcoholic etiology of cirrhosis; LD occurred in 40 patients (19.3%) and the independent predictors were Platelet-Albumin-Bilirubin Index (PALBI) grade and previous history of hepatic encephalopathy; mortality occurred in 27 patients (13%) and the independent predictors were portal vein thrombosis and MELD score. The incorporation of clinical and laboratory variables was achieved, resulting in an accurate predictive performance of ANN models with AUC ~0.84, ~0.86, and ~0.83, respectively, for OHE, LD, and mortality, emphasizing the important role of multidimensional data integration [24].

Similarly, Ince et al. [27] provided additional evidence demonstrating the ability of ML models to identify nonlinear multidimensional patterns among clinical features (e.g., TIPS indication, age, sex, cirrhosis etiology, previous HE history, ascites, CP score), biochemical (e.g., serum bilirubin, albumin, creatinine, sodium, platelets, hemoglobin, ALT, AST, INR, MELD score) and procedural features (e.g., type of stent–covered vs. bare stent—and its diameter, portal pressure pre- and post-TIPS placement, portosystemic pressure gradient) in a sample of 327 patients for predicting early post-TIPS hepatic encephalopathy within 1 month, including linear logistic regression used as baseline for comparison with other complex models, support vector machine and tree-based approaches such as random forest and gradient boosting machine. These models were trained on pre-TIPS clinical, laboratory, and procedural variables and evaluated using AUC-ROC. Ensemble models, particularly tree-based approaches, demonstrated good predictive performance with AUC between ~0.82 and ~0.84, showing higher accuracy than linear models [27].

Then, Liu et al. [32] demonstrated that supervised ML models can use clinical and biochemical variables to predict OHE occurrence and its influence on long-term survival using survival analysis (Kaplan–Meier and Cox proportional hazards models) on a sample of 218 patients who underwent TIPS placement for acute variceal bleeding. Logistic regression, random forest, and gradient boosting machine algorithms were used, trained on pre-procedural clinical (age, sex, cirrhosis etiology, previous HE history, ascites, TIPS indication, CP score, severity of acute variceal bleeding) haemodynamic parameters and laboratory (MELD score, ALT, AST, platelets, hemoglobin) independent variables selected through multivariable statistical analysis, and model performance was evaluated using AUC-ROC. OHE occurred in ~31% of patients, and post-TIPS hepatic encephalopathy showed significantly reduced survival compared to those without HE, with an approximately ~2–3-fold increased risk of mortality. Analogously to Ince et al., the highest predictive performance for OHE was obtained by tree-based models (GBM and RF), whose AUCs were ~0.86 and ~0.83, respectively, showing superiority compared to linear models (SVM and logistic regression with AUC ~0.79 and ~0.876). This study revealed HE’s central role as a determinant of long-term post-procedural prognosis, showing improved discrimination of ML models versus conventional scores, although it was a single-center study, limited by the lack of external validation [32].

In order to make the use of AI in clinical practice more accessible, practical and straightforward, and to address the difficulty in interpreting the complex set of data obtained with AI’s aid, an explainable AI (XAI) model was introduced [79,80,81]. This makes it possible to apply what has been theoretically demonstrated with the aid of artificial intelligence [82]. Ma et al. [35] implemented an XAI model to predict post-TIPS haemodynamic changes, demonstrating that interpretability tools (e.g., SHapley Additive Explanation (SHAP) values) can clarify the contribution of each patient variable, highlighting the important role of model transparency in its implementation in daily clinical practice (Ma et al.) [35].

Explainable AI can support clinician trust when it answers a specific question: which variables drive an individual prediction, whether the direction of effect is clinically plausible, and whether the model behaves consistently across relevant subgroups. SHAP is useful for global feature ranking and individual contribution plots, as shown in recent post-TIPS studies. It can also reveal data leakage or unexpected dependencies before deployment [34,43,82]. Explanation is not proof of causality or correctness. SHAP values depend on the fitted model, background distribution, and assumptions about correlated features; rankings may change after recalibration or external transport. For clinical adoption, explanations should therefore be paired with calibration-in-the-large, calibration slope, Brier score, uncertainty intervals, decision-curve analysis, subgroup performance, and explicit management thresholds. A high AUC without calibration or a defined action cannot establish clinical utility [16,17].

Song et al. [34] reported a calibrated XGBoost model interpreted with SHAP achieving good performance, but the single-center test cohort remained small.

Multicenter clinical models and mechanistically specific variables may offer more direct clinical value. A multicenter model combining clinical features with sarcopenia and post-TIPS pressure gradients improved discrimination, and portal-flow diversion on portography was associated with OHE risk. Prospective metabolomic and longitudinal microbiome studies suggest biologically plausible signals, but their sample sizes remain exploratory. The 2025 microbiome ML study illustrates the promise of high-dimensional biological data while also emphasizing the need for independent replication and standardized assays [25,26,29,30,31].

In summary, the above studies have demonstrated the ability of machine learning (ML) to improve risk stratification compared to traditional scores (e.g., Child-Pugh and MELD scores) through the dynamic, multidimensional, nonlinear integration of clinical and biochemical parameters. XAI models have shown an essential role in implementing ML in clinical practice.

The strongest conclusion from Table 1 is not a single winning algorithm. Rather, reproducible predictors cluster around age and neurological vulnerability, hepatic and renal reserve, ammonia-related metabolism, diabetes and nutritional reserve, and the magnitude or pattern of portal decompression. Model transportability depends on whether these mechanisms and their measurement are stable across indications, regions, stent practices, and prophylaxis protocols.

### 3.3. Static Models Versus Genuine Longitudinal Prediction

The term “dynamic” should not be used merely because a model is non-linear or produces an individualized probability. Almost all published post-TIPS models use a static snapshot of preprocedural variables, sometimes supplemented by immediate procedural measurements. True dynamic prediction updates risk longitudinal data accumulating and must account for measurement timing, informative missingness, recurrent events, and treatment changes.

The longitudinal microbiome study, serial metabolomics, delayed pressure-gradient work, and emerging serial biomarker studies demonstrate that trajectories may contain information not available at baseline. However, no externally validated post-TIPS Transformer, recurrent neural network, temporal convolutional network, or joint longitudinal-survival model was identified by the search cut-off. This is an evidence gap, not a reason to assume that a sequence model will outperform simpler landmark or joint models. Future comparisons should include transparent baselines, clinically realistic update times, and calibration at each landmark [29,30,82,83].

## 4. New Frontiers of Machine Learning: Radiomics and Computational Imaging in Pre-TIPS Risk Stratification

### 4.1. Complete Radiomics Workflow

A promising development in clinical practice is the integration of radiomics features into predictive models with the aid of machine learning (ML) to improve patients’ risk stratification.

A clinically credible radiomics study begins before feature extraction.

First, the prediction target, time horizon, intended use, and index time must be prespecified. Second, image acquisition variables–scanner, tube voltage/current, contrast dose and timing, slice thickness, field of view, and motion—should be documented and harmonized where possible. Third, the region of interest is segmented manually, semi-automatically, or automatically, with inter- and intra-observer reproducibility assessed when human segmentation is involved [14,15,84]. Fourth, images may require resampling, intensity discretization, normalization, denoising, bias-field correction, or registration. All preprocessing parameters must be fixed within the training data and then applied unchanged to validation data. Fifth, handcrafted first-order, shape, texture, and filtered features or learned deep features are extracted. Sixth, unstable, redundant, or weakly relevant features are reduced using reproducibility filtering, correlation pruning, penalization, or nested feature selection. Selection performed before cross-validation is a common source of information leakage [15,18,19]. Seventh, model development includes hyperparameter tuning within an inner resampling loop and unbiased performance estimation in an outer loop or, preferably, a locked temporal/geographic external cohort. Eighth, discrimination, calibration, clinical utility, and uncertainty are reported. Finally, implementation requires automated DICOM ingestion, quality control, segmentation-failure detection, processing time, output integration into the clinical record, a defined user, and prospective monitoring for drift [16,17,18,19].

### 4.2. Imaging Modalities: CT, MRI, and Ultrasound

CT dominates the direct post-TIPS evidence because contrast-enhanced CT is commonly available for preprocedural planning and simultaneously depicts hepatic and splenic morphology, portal-vein geometry, spontaneous shunts, muscle, and adipose tissue. Its main limitations are radiation, iodinated contrast, variable enhancement timing, reconstruction-kernel and slice-thickness effects, and scanner-related feature instability.

MRI offers richer tissue contrast, diffusion, perfusion, relaxometry, and brain phenotyping without ionizing radiation, but sequence heterogeneity, field strength, coil, motion, acquisition time, and access complicate harmonization. The search found contextual MRI-radiomics studies in cirrhosis and HE, but no mature externally validated MRI-radiomics model specifically for post-TIPS outcomes [59].

Ultrasound and Doppler are repeatable, bedside, inexpensive, and attractive for longitudinal monitoring of portal and shunt hemodynamics. Their limitations include operator dependence, insonation angle, body habitus, acoustic window, device settings, and lower cross-site reproducibility. Direct post-TIPS ultrasound studies support hemodynamic association and monitoring, but no validated ultrasound-radiomics prediction model was identified. This scarcity should be presented as a research opportunity, not compensated for by extrapolating CT results [85].

The comparison between different imaging modalities for multimodal post-TIPS prediction is summarized in Table 3.

### 4.3. Handcrafted Radiomics Versus Deep Radiomics

Handcrafted radiomics is relatively transparent at the feature-family level and can be used in modest datasets, but thousands of correlated features can be generated from a small number of patients. Results are highly sensitive to preprocessing, segmentation, discretization, and feature-selection strategy. Biological interpretation is often indirect.

Deep radiomics learns representations from images or segmented regions and can capture complex spatial patterns without predefining texture equations. Transfer learning may reduce sample requirements, but learned features are less interpretable, sensitive to domain shift, and vulnerable to overfitting.

Hybrid models, such as the TabPFN approach combining clinical scores, handcrafted radiomics, and deep transfer-learning features, are attractive because they integrate complementary representations; their complexity also raises the bar for independent validation, ablation analysis, calibration, and reproducible deployment [46,86].

### 4.4. Clinical Meaning of Imaging and Radiomic Features

First-order intensity features describe the distribution of attenuation or signal and may reflect enhancement, fat, edema, iron, or tissue composition. Texture and wavelet features quantify local heterogeneity and may be associated with fibrosis, nodular remodeling, perfusion heterogeneity, steatosis, inflammation, or vascular redistribution. Shape and volume features capture atrophy, hypertrophy, nodularity, and splenomegaly. Portal-vein angles and tortuosity represent flow architecture. Muscle area and density reflect nutritional and functional reserve and the capacity for extrahepatic ammonia metabolism, whereas visceral and subcutaneous fat may encode metabolic, inflammatory, and nutritional phenotypes [38,41,45,50]. These interpretations are hypotheses: a radiomic feature may change because of scanner and reconstruction settings rather than biology, and correlated features may represent the same underlying signal. Clinical translation should therefore emphasize reproducibility, robustness to protocol perturbation, feature-family consistency, and incremental predictive value rather than attaching a causal meaning to an isolated texture name.

The central question is whether imaging improves a strong clinical baseline. Several studies suggest that it can. Automated three-dimensional liver and spleen measurements improved external discrimination over clinical or 3D-only models; machine-learning-derived body composition improved MELD prediction of 90-day mortality; portal-vein geometry improved clinical models for OHE and rebleeding; and multimodal radiomics/deep-learning models reported high discrimination in external or internal validation [41,45,46,47,50].

Not all imaging additions are beneficial. A multicenter volumetry study did not find pre-TIPS liver and spleen volumes alone to be predictive, and body-composition components have shown sex-, age-, and cohort-specific associations. These negative or heterogeneous findings are scientifically important: image-derived variables should be retained only when they improve calibration or decision value in external data, not because they are technologically novel [40,48,51].

A retrospective study by Mamone et al. [42] demonstrated a correlation between pre-procedural CT radiomic features selected and analyzed by ML models, based on the acquisition of non-contrast- and portal-phase scans, and clinical outcomes after TIPS: HE (each grade), OHE ≥ grade II, six-month survival, and clinical response (absence of rebleeding and/or refractory ascites). Thus, it is reasonable to assume that imaging contains potentially prognostic information not acquired by conventional radiological assessment. Model performance was evaluated using AUC-ROC, calibration analysis, and decision curve analysis. On a sample of 76 patients, OHE occurred in ~28% of patients, and ~50–60% of them developed clinically significant OHE (≥grade II); 6-month mortality was observed in ~10% of patients and clinical response was obtained in ~75% of patients with satisfactory predictive performance (AUC ~0.9) [42].

Cheng et al. [38] developed a CT-based radiomics model with the aid of machine learning (random forest/logistic regression) that focused mainly on radiomics features of the liver for the preoperative prediction of post-TIPS HE on a sample of 106 patients, then integrated with clinical variables into a combined model. Study results showed that HE occurred in 24 patients (22.6%), and the radiomic model for HE prediction achieved good performance (AUC ~0.84) [38].

Recent evidence [87] has shown the increasing role of visceral adipose tissue (VAT) in predicting post-TIPS outcomes, particularly in cirrhotic patients undergoing TIPS placement. This expands the concept of imaging-based prognosis beyond the liver to incorporate systemic metabolic compartments [88]. In this regard, Cheng et al. [43] proposed an innovative clinical and radiomics combined approach involving the analysis of radiomic features of VAT to identify it as an emerging biomarker for predicting post-TIPS HE. In this study’s cohort, HE occurred in 63 patients in a sample of 192 (32.8%), and the model showed excellent discriminative performance (AUC ~0.9) and potential clinical utility [43].

Given the important role of radiomics as a predictive tool for post-TIPS outcomes, it is possible to state that models based on the integration of radiomics features with clinical and biochemical parameters, aided by ML, could improve prognostic accuracy. Miao et al. [46] provided one of the main examples of multimodal integration in a multicentric study with a sample of 218 patients, combining liver radiomics features, deep learning (transfer learning-derived features) and clinical scores (MELD) in a hybrid tabular prior–data fitted network (hybrid TabPFN) model to predict post-TIPS HE, which occurred in 72 patients (33%). This model achieved a very high predictive performance (AUC ~0.84) with strong external validation, demonstrating the potential of integrating heterogeneous data sources [46].

Overall, radiomics is at the forefront of post-TIPS predictive medicine, enabling the capture of complex biological information and significantly improving pre-procedural risk stratification. The predictive potential of radiomics can be further enhanced by integrating it with clinical data, but is subject to several limitations in clinical practice such as lack of external validation and limited reproducibility.

Imaging, body composition, radiomics, study design, and models’ details are summarized in Table 3.

### 4.5. Methodological Quality and Radiomics Reproducibility

IBSI standardizes feature definitions and image-processing conventions; RQS and RQS 2.0 emphasize protocol quality, robustness testing, validation, open science, and clinical utility; CLAIM and CLEAR provide reporting checklists for AI imaging and radiomics studies. A post-TIPS radiomics study should report scanner and acquisition parameters, contrast phase, segmentation method and reproducibility, preprocessing, software and feature definitions, missing data, sample and event counts, full resampling strategy, calibration, external validation, and availability of code or a locked model, and the existing literature rarely satisfies all of these criteria [14,15,18,19].

External multicenter validation has improved in recent studies, but many models remain internally split, and performance is usually reported as AUC without full calibration. Robustness to scanner, phase, slice thickness, and segmentation perturbation is inconsistently documented. This limits reproducibility and makes high apparent AUCs insufficient evidence for clinical deployment.

## 5. Limitations of Artificial Intelligence, Critical Appraisal of the Evidence, and Future Perspectives

### 5.1. Comparative Performance and Clinical Applicability

Clinical and biochemical models have the lowest implementation burden and are easiest to audit, but they may omit anatomic and tissue-level information. Procedural/hemodynamic variables are mechanistically informative and potentially actionable, although some become available only after the procedure has started. Body-composition and conventional imaging variables can be added to routine CT with modest workflow burden, particularly when segmentation is automated. Handcrafted radiomics and deep features may improve discrimination, but they add the greatest risks of protocol sensitivity, leakage, and model opacity. These statements are summarized in Table 4.

### 5.2. Common Sources of Bias and Limited Generalizability

Despite promising results, the clinical application of ML in the TIPS setting is still hindered by several significant limitations. The first of these is the studies’ designs and characteristics. Most of the available studies are retrospective and single-center [24,27,32,43], which limits the generalisability of their findings. The only prospective study is that by Ma et al. [35], which is an exception in the current literature. Sample sizes are frequently modest relative to the number of candidate predictors, especially for radiomics. Another important limitation is the heterogeneity of the study populations: populations differ in TIPS indication, cirrhosis etiology, stent type and diameter, embolization practice, prophylaxis, transplant availability, imaging data, and outcome adjudication. OHE definitions and follow-up windows vary and patients are often highly selected, probably introducing bias and limiting the applicability of the findings more broadly. External validation, which is crucial to guaranteeing the reliability and reproducibility of predictive models, is often lacking. This creates a significant bias in the application of study findings in clinical practice.

A relevant methodological limitation is the insufficient assessment of model transportability beyond the development setting. The lack of locked external temporal or geographic validation, together with limited evaluation of calibration, decision-curve analysis, and subgroup performance, prevents reliable estimation of whether current post-TIPS prediction models can maintain performance across different populations, clinical practices, and evolving TIPS strategies.

From a technical standpoint, even the study by Miao et al. [46], which demonstrated the most accurate models (e.g., deep learning and hybrid clinical and radiomics approaches) for predicting outcomes, is associated with several limitations. These include the difficulty of interpreting data due to its high computational complexity and the challenge of integrating it into clinical workflows. Although explainable AI approaches represent a crucial step forward, achieving a balance between the performance of predictive tools and interpretability remains an ongoing challenge.

Finally, radiomics is burdened by standardization issues. First, there is a lack of standardized processes for identifying and extracting features. Second, there is variability in CT acquisition protocols. Third, there is a necessity for highly skilled, dedicated specialists. Therefore, future research should prioritize the following: the creation of prospective multicenter studies with homogeneous population selection and external validation; the development of interpretable, user-friendly hybrid models; the optimization of explainable AI models; and the standardization of radiomics methodologies.

### 5.3. Future Perspectives

Future studies should prospectively recruit a larger and more homogeneous number of consecutive patients across countries and center types in prospective multicenter studies with standardized selection criteria of inclusion, external validation, rigorous calibration assessment, and transparent reporting to ensure generalizability and facilitate clinical translation.

A core data set should include cognition, frailty, medications, serial ammonia with preanalytic metadata, renal and liver tests, hemodynamics, routine DICOM imaging, body composition, and patient-reported outcomes.

Future research should prioritize the development and external validation of Transformer-based models capable of capturing longitudinal, multimodal post-TIPS data. The identification of robust and externally validated Transformer architectures may enhance the scientific validity and clinical reliability of future studies by improving the modeling of dynamic patient trajectories, reducing dependence on single preprocedural snapshots, and enabling more accurate prediction of long-term post-TIPS outcomes.

## 6. Conclusions

Machine learning is a promising tool for predicting post-TIPS outcomes, especially hepatic encephalopathy. Multidimensional, dynamic models based on clinical and biochemical variables have demonstrated promising predictive performance, which could be further improved by integrating radiomics to create valid, multimodal approaches. However, the clinical implementation of these tools is currently limited by methodological issues, including a lack of external validation, the prevalence of retrospective study designs, and model complexity.

In conclusion, while the study’s results appear very promising, further methodological development and clinical validation are necessary before artificial intelligence can be incorporated into the clinical management of patients undergoing TIPS placement.

## Figures and Tables

**Table 1:** Clinical, biochemical, procedural, hemodynamic, microbiome, and metabolomic studies included in the core evidence set (n = 18). AUC values are reported as presented by the original studies and are not directly comparable because outcome horizons and validation designs differ.

Study/Design	Target	Input Domains	Method and Validation	Principal Finding	Critical Appraisal
Nardelli et al., 2016 [20] Prospective cohort	Post-TIPS HE	Cognition; clinical; laboratory	Multivariable analysis; single center	Pre-TIPS cognitive impairment predicted HE.	Clinically intuitive; modest sample; no external validation.
Yin et al., 2020 [21] Retrospective n = 373	1-y OHE-free survival	Age, CTP, diabetes, creatinine, sodium	Cox nomogram; 70/30 random split	C-index about 0.77; AUC about 0.81/0.78.	Accessible variables; random split and single-center optimism.
Yang et al., 2021 [22] Retrospective n = 185	Post-TIPS OHE	Clinical plus imaging characteristics	Logistic regression; random train/validation split	Combined model outperformed clinical-only and imaging-only models.	Very high internal AUC; no independent external cohort.
Tong et al., 2021 [23] Derivation n = 299; validation n = 62	Post-TIPS OHE	Etiology, stent diameter, PPG reduction, MELD, ammonia, hydrothorax	Logistic risk score; separate temporal/center validation cohort	Sensitivity and specificity about 71%.	Transparent and actionable; thresholds may not transfer.
Zhong et al., 2021 [24] Retrospective n = 207	3-mo OHE; liver dysfunction; 1-y mortality	Clinical and laboratory	ANNs versus regression nomograms; internal validation	AUCs approximately 0.83–0.86 across outcomes.	Multi-outcome study; single center, no independent external test.
Yang et al., 2022 multicenter [25]	3-mo OHE	Clinical, sarcopenia, post-TIPS PPG	Regression models; external multicenter validation	Adding sarcopenia and PPG improved C-index above the core clinical model.	Important incremental-value analysis; some predictors become available only during/after TIPS.
Yang et al., 2022 portography [26]	Post-TIPS OHE	Portal-flow diversion; ammonia; clinical	Competing-risk/Cox analysis; single center	SMV-dominant diversion was associated with higher OHE risk.	Mechanistically plausible and potentially actionable; operator- and technique-dependent.
Ince et al., 2023 [27] Retrospective n = 327	Early OHE	Clinical, laboratory, procedural	LR, SVM, CatBoost; nested 5-fold CV	Non-linear models achieved test AUCs about 0.82–0.83.	Methodologically stronger internal validation; no geographic external validation.
Liao et al., 2023 [28] Retrospective n = 296	Post-TIPS OHE	Age, ammonia, creatinine, ICG-R15, PPG reduction	LASSO/regression nomogram; random split	C-index/AUC about 0.83 in internal validation.	Includes functional liver testing; single-center and split-sample limitations.
Machado et al., 2023 [29] Prospective n = 22	HE severity over 1 y	Untargeted plasma metabolomics; shunting phenotype	Exploratory longitudinal analysis	Bile-acid and phospholipid signatures varied with HE severity.	Biologically rich but very small; hypothesis-generating, not deployment-ready.
Li et al., 2022 [30] Prospective n = 106	6-mo HE and survival	Serial 16S microbiome; clinical	Longitudinal association models	Microbiota restoration was associated with lower HE risk.	True longitudinal sampling; not a locked externally validated clinical model.
Li et al., 2025 [31] Metagenomic n = 67	HE and ammonia change	Metagenomics; pathways; clinical	Multiple ML models; internal evaluation	Microbiome features, including *P. vulgatus*, showed predictive signal.	High dimensionality with very small sample; substantial overfitting risk.
Liu et al., 2025 [32] Retrospective n = 218	OHE and long-term survival after AVB	Clinical, biochemical, hemodynamic	LR, RF, GBM; internal testing	Tree-based models showed good OHE discrimination; OHE related to survival.	Selected acute-variceal-bleeding population; no external validation.
Jiang et al., 2025 [33] Retrospective n = 440	1-y OHE	Preoperative clinical and nutritional variables	LASSO plus logistic nomogram; 7/3 split	Good discrimination, calibration, and decision curves internally.	Large single-center cohort; random split is not independent transport validation.
Song et al., 2026 [34] Retrospective n = 297	1-y OHE	BUN, GGT, age, fibrinogen, puncture site	LR, SVM, RF, XGBoost, ANN; 8/2 split; calibration; SHAP	XGBoost test AUC 0.792.	Broad model comparison; small test set and single-center practice.
Ma et al., 2026 [35] Retrospective n = 328; prospective n = 128	Delayed PPG change and further decompensation	Immediate hemodynamics and clinical features	SVR-RBF; 5-fold CV; prospective validation; SHAP	Improved prediction of delayed PPG and prognostic stratification.	Uncommon prospective validation; surrogate hemodynamic target requires workflow integration.
Spaan et al., 2026 [36] Retrospective n = 325	HE and severe HE	Diabetes, age, MELD-Na, clinical	Competing-risk Cox models	Diabetes independently associated with HE and hospitalization.	Strong clinical signal; association study rather than a complete prediction model.
Xiang et al., 2025 [37] Large multicenter real-world cohort	Short- and long-term mortality after OHE	Clinical and outcome trajectories	Propensity methods and competing-risk analysis	OHE associated with long-term, not early, mortality.	Clarifies target impact and time horizon; not a pre-TIPS model-development study.

**Table 2:** Imaging, body-composition, radiomics, and multimodal studies included in the core evidence set (n = 14).

Study/Sample	Modality and ROI	Segmentation/Feature Type	Model and Validation	Performance/Incremental Value	Key Limitation
Cheng et al., 2022 [38] n = 106	Pre-TIPS CT; liver	Manual liver segmentation; handcrafted radiomics	Feature selection plus ML; internal validation	Radiomics AUC about 0.80; combined model about 0.84.	Small single-center cohort; no external validation.
Cai et al., 2022 [39]	Unenhanced CT at L3; psoas muscle	Manual psoas density	Regression; single center	Lower psoas density associated with 1-y HE.	Single-slice biomarker; manual measurement; no external test.
Wang et al., 2023 [40]	CT at L3; VAT, SAT, muscle	Manual/semi-automatic body composition	Sex-specific regression nomograms; internal validation	Low VAT index in men and low SAT index in women predicted HE.	Sex-specific cutoffs and protocol dependence limit transport.
Chen et al., 2023 [41] n = 487, 5 hospitals	Contrast CT; liver and spleen	Automatic 3D segmentation; 2D/3D measurements	LASSO, SVM, isotonic calibration; external validation	Combined clinical+3D AUC 0.816 externally versus 0.723 clinical.	External sample still modest; dependence on segmentation pipeline.
Mamone et al., 2024 [42] n = 76	Noncontrast and portal-phase CT; liver	Manual ROI; handcrafted radiomics	LR/SVM/RF; internal validation; calibration/DCA	Combined models showed promising AUCs across HE, survival, and response.	Multiple outcomes in a very small cohort; high optimism risk.
Cheng et al., 2024 [43]	Pre-TIPS CT; visceral adipose tissue	VAT segmentation; first-order, texture, wavelet	LR/SVM/RF; external testing	Radiomic VAT features added predictive information for HE.	Feature stability and segmentation transfer require confirmation.
Chen et al., 2024 [44] n = 621	Contrast CT/CTA; vascular morphology	Vascular and clinical measurements	Multicenter model with external validation	Good discrimination (reported AUC/C-index around 0.78–0.81).	Morphologic definitions and acquisition protocols may vary by center.
Elhakim et al., 2025 [45] n = 122 multicenter	Contrast CT at L3; muscle and fat	Automated ML-derived body composition	Logistic models; multicenter retrospective cohort	MELD plus SMA/SFA/VFA AUC 0.84 versus MELD 0.76.	CT-to-TIPS interval up to 9 months; small mortality event count.
Miao et al., 2026 [46] n = 218, 3 hospitals	CECT; liver, spleen, fat, muscle	Radiomics plus deep transfer-learning features	Hybrid TabPFN + MELD; external validation; SHAP	External AUC reported 0.942.	Small external cohort; complex pipeline and potential optimism.
Zhou et al., 2026 [47] n = 338	Pre-TIPS CT; manual imaging and radiomics	Manual features plus radiomics	Multimodal ML; internal 7/3 split	Combined validation AUC about 0.91.	No external validation; split-sample feature-selection risk.
Saltini et al., 2026 [48] n = 115, age ≥ 70 y	Routine CT at L3	Sarcopenia and myosteatosis	Multicenter Cox/time-dependent ROC	Adding sarcopenia improved MELD 3.0 mortality AUC to 0.845.	Older-adult subgroup; not a general-population model.
Li et al., 2026 [49] n = 239	Pre-TIPS CT at L3; VAT and SAT	Area measurements and V/S ratio	ROC and Cox models; retrospective	V/S ratio AUC 0.817 for 1-y OHE.	Single biomarker and no independent external validation.
Wan et al., 2026 [50] n = 579 multicenter	CTA; 3D portal vein geometry	Automated/quantitative angles and tortuosity	Cox models; multicenter validation	Clinical+geometry C-index about 0.80 for OHE and 0.79 for rebleeding.	Requires robust vascular segmentation and protocol transfer.
Bedoya et al., 2026 [51] n = 160, 3 centers	CT/MRI; liver and spleen volume	Volumetry	Multicenter association/prediction analysis	Volumes alone did not predict post-TIPS outcomes.	Important negative study; does not exclude value of texture or geometry.

**Table 3:** Comparison of imaging modalities for multimodal post-TIPS prediction.

Modality	Potential Predictive Information	Advantages	Reproducibility Threats	Current Post-TIPS Evidence
CT/CTA	Liver and spleen morphology; portal geometry; spontaneous shunts; muscle and fat; handcrafted/deep radiomics	Routine planning study; fast; whole-abdomen coverage; scalable automation	Contrast phase, scanner, kernel, slice thickness, radiation, segmentation	Most developed; several multicenter/external studies, but heterogeneous methods.
MRI	Parenchymal composition, diffusion, perfusion, fibrosis surrogates, brain changes	Multiparametric contrast; no ionizing radiation	Sequence and field-strength heterogeneity; motion; longer acquisition; access	Direct post-TIPS radiomics evidence remains sparse; mostly contextual HE work.
Ultrasound/Doppler/elastography	Flow velocity/direction, shunt patency, portal and arterial dynamics, stiffness	Bedside, repeatable, low cost, suited to serial monitoring	Operator, angle, acoustic window, device presets, limited retrospective standardization	Association and monitoring studies exist; no validated post-TIPS ultrasound-radiomics model identified.

**Table 4:** Qualitative level-of-evidence and translation-readiness assessment. The categories are narrative judgments, not formal GRADE ratings.

Evidence Domain	Current Strengths	Dominant Limitations	Overall Evidence Level	Priority Before Deployment
Clinical/biochemical regression and nomograms	Accessible, transparent, repeated predictors, several moderately sized cohorts	Retrospective design, heterogeneous endpoints, random split validation	Moderate for association; low-to-moderate for transportable individualized prediction	External recalibration and head-to-head comparison with routine scores.
Tree ensembles/SVM/ANN on tabular data	Can model non-linearity and interactions; some nested CV and prospective validation	Small samples, limited external validation, variable calibration	Low-to-moderate	Locked multicenter validation, calibration, and clinical-impact testing.
Hemodynamic/procedural prediction	Mechanistically close to intervention and potentially actionable	Timing may be intra/post-procedural; measurement and technique variability	Moderate for selected variables	Prospective decision rules linked to stent/PPG strategies.
Body composition and conventional imaging	Uses routine CT; plausible biology; some multicenter incremental-value studies	Cutoff heterogeneity, sex/age effects, segmentation and CT timing	Moderate for sarcopenia/body composition associations; lower for universal thresholds	Automated standardized measurement and external threshold validation.
Handcrafted radiomics	Captures texture and heterogeneity beyond visual assessment	Feature instability, leakage, small event counts, scarce external validation	Low	IBSI-compliant pipelines, robustness testing, code/model sharing, external validation.
Deep/multimodal radiomics	High reported discrimination; integration of complementary data	Complexity, opacity, small external cohorts, domain shift	Low-to-moderate but rapidly evolving	Large geographic external validation, ablation studies, calibration, workflow and impact trials.
Longitudinal/sequence prediction	Biologically plausible and suited to changing risk	Very limited direct post-TIPS model evidence; no validated Transformer/sequence model	Very low/evidence gap	Prospective serial data, transparent landmarks, joint models, and fair comparison with simpler baselines.

## Data Availability

No new data were created or analyzed in this study. Data sharing is not applicable to this article.
